# Delineating structural and metabolic abnormalities in amygdala and hippocampal subfields for different seizure‐onset patterns via stereotactic electroencephalography

**DOI:** 10.1111/cns.14905

**Published:** 2024-09-09

**Authors:** Tao Feng, Yanfeng Yang, Yihe Wang, Peng‐hu Wei, Xiaotong Fan, Huaqiang Zhang, Yang An, Tianren Wang, Yuda Huang, Sichang Chen, Yueshan Piao, Fenglai Xiao, John S. Duncan, Yongzhi Shan, Guoguang Zhao

**Affiliations:** ^1^ Department of Neurosurgery Xuanwu Hospital, Capital Medical University Beijing China; ^2^ China International Neuroscience Institute (CHINA‐INI) Beijing China; ^3^ Department of Clinical & Experimental Epilepsy UCL Queen Square Institute of Neurology London UK; ^4^ Department of Pathology, Xuanwu Hospital Capital Medical University Beijing China; ^5^ MRI Unit, Chalfont Centre for Epilepsy Chalfont Saint Peter UK; ^6^ Institute for Brain Disorder Beijing China

**Keywords:** amygdala, hippocampus, seizure onset patterns, stereotactic electroencephalography, temporal lobe epilepsy

## Abstract

**Aims:**

We aimed to investigate mesial temporal lobe abnormalities in mesial temporal lobe epilepsy (MTLE) patients with hypersynchronous (HYP) and low‐voltage fast rhythms (LVF) onset identified by stereotactic electroencephalography (SEEG) and evaluate their diagnostic and prognostic value.

**Methods:**

Fifty‐one MTLE patients were categorized as HYP or LVF by SEEG. High‐resolution MRI volume‐based analysis and ^18^F‐FDG‐PET standard uptake values of hippocampal and amygdala subfields were quantified and compared with 57 matched controls. Further analyses were conducted to delineate the distinct pathological characteristics differentiating the two groups. Diagnostic and prognostic prediction performance of these biomarkers were assessed using receiver operating characteristic curves.

**Results:**

LVF‐onset individuals demonstrated ipsilateral amygdala enlargement (*p* = 0.048) and contralateral hippocampus hypermetabolism (*p* = 0.042), pathological results often accompany abnormalities in the temporal lobe cortex, while HYP‐onset subjects had significant atrophy (*p* < 0.001) and hypometabolism (*p* = 0.013) in ipsilateral hippocampus and its subfields, as well as amygdala atrophy (*p* < 0.001), pathological results are highly correlated with hippocampal sclerosis. Severe fimbria atrophy was observed in cases of HYP‐onset MTLE with poor prognosis (AUC = 0.874).

**Conclusion:**

Individuals with different seizure‐onset patterns display specific morphological and metabolic abnormalities in the amygdala and hippocampus. Identifying these subfield abnormalities can improve diagnostic and prognostic precision, guiding surgical strategies for MTLE.

## Graphical abstract

Hypersynchrony (HYP) and low‐voltage fast (LVF) rhythm are the two most common forms of onset in mesial temporal lobe epilepsy (MTLE). HYP onset often involve atrophy and hypometabolism of the ipsilateral hippocampus and amygdala, while LVF onset is mainly characterized by hypertrophy of the amygdala. Different SOPs also involve different pathology.

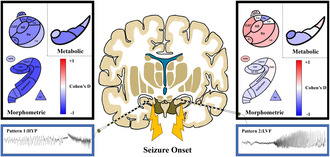

## 1 INTRODUCTION

Mesial temporal lobe epilepsy (MTLE) is the most common type of medically intractable focal epilepsy.^1^ Hypersynchronous (HYP) and low‐voltage fast (LVF) rhythms are the two main types of MTLE seizure onset identified with stereotactic electroencephalography (SEEG).^2^, ^3^, ^4^ These two seizure‐onset patterns (SOPs) have differing pathogenesis, seizure duration, propagation time, distribution,^4^, ^5^, ^6^ and surgical prognoses.^7^, ^8^, ^9^

Onset with LVF is more frequently observed in neocortical epilepsy and focal cortex dysplasia (FCD), whereas HYP onset is only observed in MTLE.^4^, ^10^ The hippocampi of patients with HYP‐onset MTLE have typical hippocampal neuronal loss and atrophy, but patients with LVF‐onset MTLE do not show these pathological alterations.^9^ Thus, HYP onset implies injury to mesial temporal lobe structures.^11^ In contrast, LVF onset in MTLE may imply extrahippocampal seizures.^12^, ^13^

In keeping with the pathology, individuals with HYP‐onset MTLE have a better surgical prognosis than those of LVF,^7^, ^8^ suggesting that these individuals may require distinct treatment approaches. Clarifying the morphological and metabolic abnormalities of the medial temporal lobe structures associated with these two SOPs is important for optimizing treatment. Magnetic resonance imaging (MRI) has revealed that the hippocampal atrophy (HA) of HYP‐onset MTLE was concentrated in the medial hippocampus, while the HA of LVF‐onset MTLE is distributed in the lateral hippocampus,^13^ indicating that patterns of HA may differ at the subfield level. The differences in morphological and metabolic sub‐regional patterns in the medial temporal lobes between the two groups of patients, and the prognostic significance of these are not clear.

## 2 MATERIALS AND METHODS

Between May 2016 and August 2022, we recruited patients who underwent SEEG in the Department of Neurosurgery at Xuanwu Hospital Capital Medical University, Beijing, and had drug‐resistant focal epilepsy that was considered to originate from the medial temporal lobe. We selected healthy controls (HCs) using structural MRI scans from Xuanwu Hospital for the morphological analysis and selected the positron emission tomography (PET)/MR data of HCs from the OpenNeuro Dataset ds004513 (https://openneuro.org/datasets/ds004513) for the metabolic analysis.^14^

The inclusion criteria for the patient cohort were: (1) structural MRI at Xuanwu Hospital; (2) SOPs recorded by SEEG were either HYP or LVF; (3) at least one habitual seizure was recorded using SEEG, and the origin of the mesial temporal lobe was confirmed; (4) radiofrequency thermocoagulation (RFTC) or anterior temporal lobe resection (ATLR) was performed at Xuanwu Hospital.

The exclusion criteria were: (1) no habitual episode was detected using SEEG; (2) SEEG‐recorded seizures were only induced using electrical stimulation; (3) SEEG recorded the simultaneous existence of two SOPs; and (4) preoperative scalp electroencephalography (EEG), structural MRI, ^18^F‐fluorodeoxyglucose (^18^F‐FDG) PET, SEEG recording, and postoperative pathology indicated the possibility of bilateral MTLE.

The volume and standard uptake value (SUV) of ^18^F‐FDG in each subfield of the amygdala and hippocampus were primary observational markers. All patients provided written informed consent, and the Xuanwu Hospital Capital Medical University Ethics Committee authorized all procedures.

### 2.1 SEEG implantation and reconstruction

All patients enrolled in the study had at least one intracerebral electrode in the hippocampus. SEEG implantation (Alcis, Besancon, France) was performed using a ROSA robot system (Medtech, Montpellier, France) based on preoperative three‐dimensional T1‐weighted contrast MRI. SEEG electrodes had 5–18 contacts, each measuring 0.8 mm in diameter and 2 mm in length, with 1.5 mm spacing between them. Each patient underwent postoperative computed tomography (CT) to reconstruct the SEEG electrodes.

**FIGURE 1:**
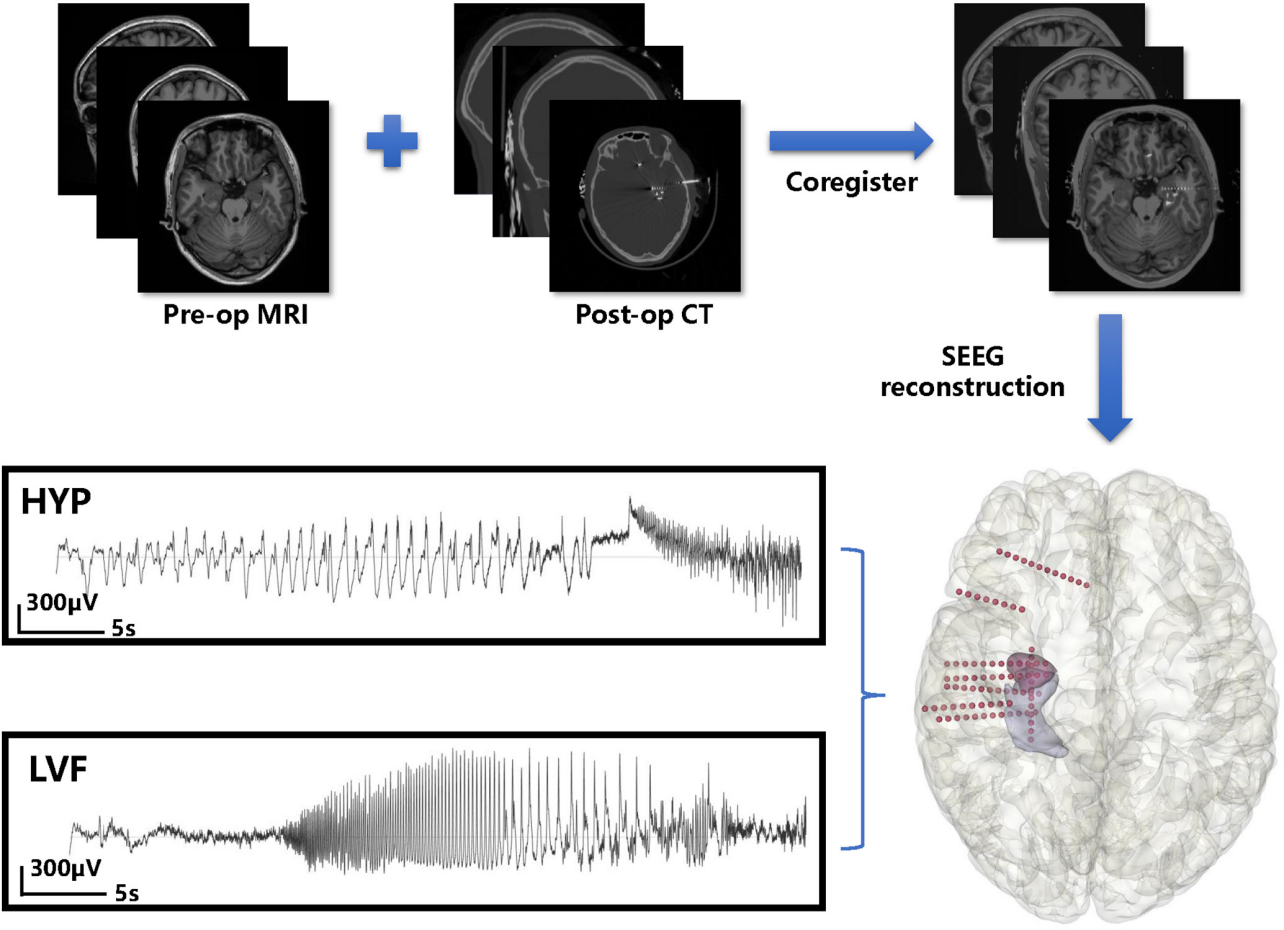
SEEG reconstruction and seizure onset pattern classification. Preoperative MRI and postoperative CT were used to perform SEEG reconstruction. SEEG implanted in the medial temporal lobe distinguished two different SOPs. SOP labeled HYP showed high amplitude spikes <2 Hz that lasted for ≥5 s, while labeled LVF showed low amplitude and high frequency (≥10 Hz) activity. HYP, hypersynchronous; LVF, low‐voltage fast rhythms.

Postoperative video SEEG monitoring was performed in all patients to identify the seizure onset zone (Figure 1). The Nicolet system (Natus Medical, San Carlos, CA) was used to capture the SEEG data at a 2048‐Hz sampling rate.

### 2.2 SOP classification

The LVF SOP displayed low amplitude and high frequency (≥10 Hz) activity, while the HYP SOP displayed rhythmic, high amplitude spikes <2 Hz that lasted for ≥5 s^15^, ^16^ (Figure 1).

### 2.3 Structural MRI and ^18^F‐FDG‐PET data acquisition

Structural MRI of the patient cohort and HCs was performed using a GE Premier 3.0‐Tesla MRI scanner (GE Healthcare, Waukesha, WI) with a 64‐channel head–neck coil at Xuanwu Hospital Capital Medical University using a T1‐weighted magnetization‐prepared rapid gradient‐echo sequence. The structural MRI parameters were as follows: voxel size: 1.0 × 1.0 × 1.0 mm^3^, field of view: 256 × 256 mm, repetition time: 2476 ms, time to echo: 2.7 ms, inversion time: 0.9 s.

A uMI PET/CT scanner (United Imaging Healthcare, Shanghai, China) was used to obtain the ^18^F‐FDG‐PET images of the patient cohort. Patients were instructed to fast for at least 6 h and then receive an intravenous injection of 3.7 MBq/kg of ^18^F‐FDG. PET images were obtained after a calm rest period of 30 min. Reconstructed images of ^18^F‐FDG‐PET had a voxel size of 1.82 × 1.82 × 2.78 mm^3^ and a matrix of 192 × 192 × 89.

We acquired ^18^F‐FDG‐PET images of the HCs using a Siemens Biograph mMR 3.0‐T PET/MR scanner from the OpenNeuro Dataset ds004513.^14^ The ^18^F‐FDG‐PET images were reconstructed in a 344 × 344 × 127 matrix with a voxel size of 2.08 × 2.08 × 2.03 mm^3^.

### 2.4 Segmentation of individualized amygdala and hippocampal subfields

We employed FreeSurfer version 7.0 to perform quantitative automated volumetric segmentation of T1‐weighted MRI for each participant, including HCs and patients, to gather individual brain area morphological data and the estimated total intracranial volume (eTIV). On this basis, we used the FreeSurfer‐based approach outlined by Iglesias and Saygin to perform adaptive labeling and segmentation of the hippocampal and amygdala subfields.^17^, ^18^ We segmented 12 subfields of the hippocampal region automatically, including the CA1–4, subiculum, dentate gyrus (DG), hippocampus‐amygdala‐transition‐area (HATA), molecular layer (ML), and fimbria, and nine subfields of the amygdala, including the lateral nucleus (La), basal nucleus (Ba), accessory‐basal nucleus (AB), anterior‐amygdala‐area (AAA), central nucleus (Ce), medial nucleus (Me), cortical nucleus (Co), corticoamygdaloid‐transition (CAT), and paralaminar nucleus (PL). The segmentation results were manually corrected for errors, including non‐brain structures, and the volume of each subfield was determined.

We corrected the volume of each subfield using the eTIV as follows: volume _cor_ = volume _subject_ ×1000/eTIV _subject_, to obtain the corrected volume for each hippocampal and amygdala subfield for each patient.

### 2.5 Glucose metabolism in amygdala and hippocampal subfields

We co‐registered ^18^F‐FDG‐PET image data of the patients with individual T1‐weighted MRI using the 4th‐degree B‐spline and the Normalized Mutual Information function of SPM12. Given the special resolution and full‐width half‐maximum characteristics of ^18^F‐FDG‐PET, quantifying the SUV of the amygdala and hippocampal subfields is susceptible to partial volume effects. To address this, we employed Freesurfer for segmenting the hippocampus into three distinct subfields based on T1‐weighted MRI, including head, body, and tail (HBT subfield). We then utilized individual amygdala and hippocampal HBT subfield masks to facilitate precise extraction of the SUV for each subfield using the Data Processing & Analysis for Brain Imaging (DPABI) toolkit^19^ (Figure S1). To reduce inter‐participant differences and discrepancies between PET machines and scanning sequences, we calculated SUV ratios (SUVR) using the SUV of the cerebellum with the formula SUVR_hip/amy_ = SUV_hip/amy_/SUV_cerebellum_. We therefore obtained the corrected SUVR value of each subfield for each patient.

### 2.6 Pathological verification of two SOPs for MTLE

To more comprehensively compare the pathological underpinnings of morphological and metabolic discrepancies between the two groups, we amassed histological specimens from surgical resections. These specimens were then subjected to a detailed evaluation of their histological features and neuronal depletion within the resected hippocampus, utilizing both hematoxylin and eosin staining and NeuN immunohistochemical staining techniques. In instances of unique pathological findings, targeted immunohistochemical staining approaches were employed to ascertain the definitive pathological outcomes. Subsequently, we conducted a comparative analysis of the hippocampal pathological characteristics and the ultimate pathological findings across the groups.

### 2.7 Surgical outcome assessment

Surgical outcomes were assessed via telephone and outpatient follow‐up using the International League Against Epilepsy (ILAE) grading system with a minimum follow‐up time of more than 12 months. The ILAE classification is defined as complete seizure freedom without auras (class 1), only auras without other seizures (class 2), 1–3 seizure days/year (class 3), 4 seizure days/year to a 50% decrease from pre‐surgical seizure frequency (class 4), between a 50% and 100% increase (class 5), and ≥100% increase in seizure frequency (class 6).^20^

### 2.8 Statistical analysis

All data were analyzed using the Statistical Package for the Social Sciences (version 22; IBM, Armonk, NY). All data were verified by the Kolmogorov–Smirnov test with Dallal‐Wilkinson‐Lillie for *p* value, and the data that did not meet the normal (Gaussian) distribution were verified by the non‐parametric test. The clinical characteristics of the two groups were compared using the independent‐samples *t*‐test, Mann–Whitney *U* test, and Kolmogorov–Smirnov test.

Patients' amygdala and hippocampal subfields within the hemisphere of seizure onset were labeled “ipsilateral,” and the amygdala and hippocampal subfields within the hemisphere opposite the side of seizure onset were labeled “contralateral.” In addition, since it was not possible to flip the HCs' data to match the ipsilateral and contralateral sides in patients, we first calculated the z‐scores for all patients using the following formula: Zpat = (volume/SUVRpat – μHC)/σHC, where μHC and σHC represent the mean and standard deviation of the volume or SUVR of the HCs, for each patient at each hemisphere, and we flipped the *z*‐scores of right MTLE patients afterwards to ensure that the left side of MTLE patients and HCs corresponded to the ipsilateral side and the right side to the contralateral side.^21^

To compare the differences in the volume and SUVR values of the bilateral amygdala and hippocampal subfields in patients with MTLE with two SOPs and those in HCs, one‐way analysis of covariance corrected for age and sex was used to compare the volumes and SUVR z‐scores of the ipsilateral and contralateral hippocampal and amygdala subfields between the three groups (HYP, LVF, and HCs). False discovery rate (Benjamin–Hochberg) correction was used for multiple comparisons.

To determine whether there are differences in surgical outcomes between MTLE patients with different SOPs, we used the Mann–Whitney *U* test to compare the ILAE grades between the two groups under different surgical approaches. Further, to determine whether atrophy and hypometabolism based on amygdala and hippocampal subfields can improve MTLE diagnosis accuracy, a descriptive analysis of the sensitivity and specificity between the subfields were compared in MTLE diagnosis using receiver operating characteristic (ROC) curves. To identify the most valuable indicator of surgical prognosis, we used ROC curves to compare the sensitivity and specificity of different atrophy and hypometabolism subfields on MTLE surgery prognosis.

## 3 RESULTS

Fifty‐one individuals with MTLE (28 males; aged 12–55 years; mean 28.6 ± 8.1 years) and 37 HCs with structural MRI (19 males; aged 21–30 years; mean 26.4 ± 2.4 years) and 20 HCs with ^18^F‐FDG‐PET/MR (10 males; aged 22–52 years; mean 34.5 ± 10.0 years) were included in this study. Based on SEEG results, the patient cohort was divided into the HYP (24 patients; 10 males; aged 12–55 years; mean 29.2 ± 9.2 years) and LVF (27 patients; 18 males; aged 18–49 years; mean 28.1 ± 7.2 years) groups (Table 1). The ^18^F‐FDG‐PET data of one patient in the HYP group and two patients in the LVF group were excluded for quality control reasons.

**TABLE 1:** Clinical and demographic characteristics of study cohort.

	HYP group	LVF group	*p*	Healthy controls (MRI group)	Healthy controls (PET group)
Total number	24	27	–	37	20
Age (mean ± SD)	12–55	18–49	0.637	21–30	22–52
	29.2 ± 9.2	28.1 ± 7.2		26.4 ± 2.4	34.5 ± 10.0
Sex					
Male	10	18	0.827	19	10
Female	14	9		18	10
Duration of epilepsy (years)	14.1 ± 10.1	12.6 ± 8.3	0.582	–	–
Lateralization					
Left	13	15	>0.999	–	–
Right	11	12		–	–
Frequency of focal seizures (*n*/month)	5.8 ± 7.0	15.0 ± 20.9	0.044*	–	–
Surgery					
ATLR	3	11	0.263	–	–
RFTC	21	16		–	–
Pathology					
HS	3	1	–	–	–
DNT	0	1			
Hip gliosis + mMCD	0	4			
MOGHE	0	1			
Hip gliosis	0	4			
Prognosis					
ILAE 1	17	12	0.048*	–	–
ILAE 2–6	7	15		–	–

*Note*: Continuous variables were presented as mean ± SD. Independent samples *t* tests were used for continuous variables, and Mann–Whitney *U* tests or Kolmogorov–Smirnov tests were used for categorical variables, the '*' indicates the significant result of the statistical analysis (*p* < 0.05)

Abbreviations: ATLR, anterior temporal lobe resection; DNT, dysembryoplastic neuroepithelial tumor; HS, hippocampal sclerosis; HYP, hypersynchronous; LVF, low‐voltage fast rhythms; mMCD, mild malformation of cortical development; MOGHE, mild malformation of cortical development with oligodendroglial hyperplasia in epilepsy; RFTC, radiofrequency thermocoagulation.

### 3.1 Morphological analysis of the amygdala and hippocampal subfields

The HYP‐onset group had significantly decreased ipsilateral amygdala (HYP z‐scores: −1.182 ± 1.427, *p* < 0.001) and hippocampal volume (HYP *z*‐scores: −2.300 ± 1.211, *p* < 0.001). Conversely, amygdala volume in the LVF‐onset group was significantly increased (LVF z‐scores: 0.722 ± 1.540, *p* = 0.048), whereas hippocampal volume was not significantly different from that in the HCs (LVF *z*‐scores: −0.388 ± 1.361, *p* = 0.552).

**FIGURE 2:**
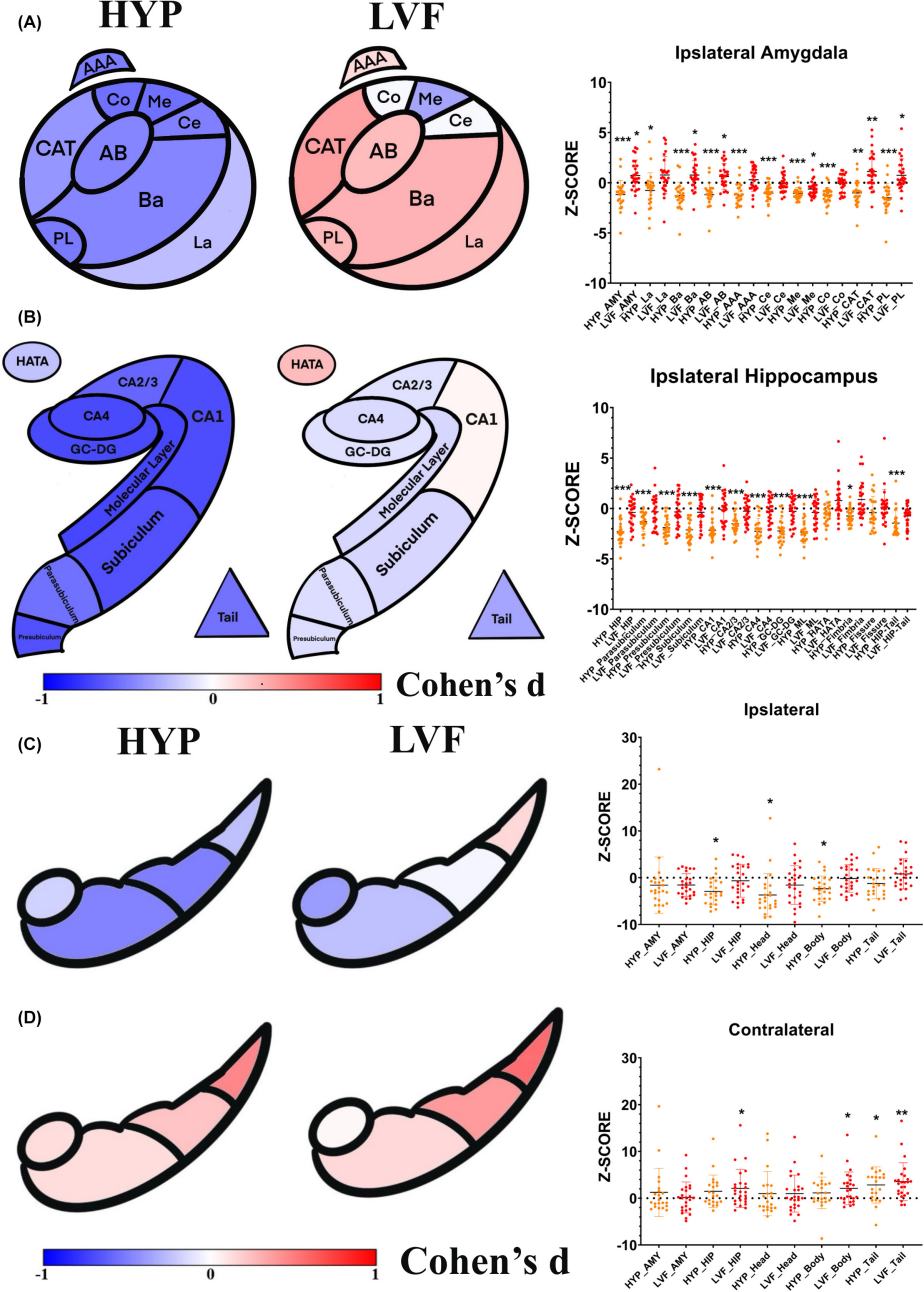
Volumetric and metabolic analysis of the ipsilateral amygdala and hippocampal subfields between patients and HCs. The comparative analysis of the volumes of ipsilateral amygdala subfields between the HYP‐onset group and HCs, as well as between the LVF‐onset group and HCs, is quantified using Cohen's d effect size values. A value approaching −1 indicates a reduction in the volume of amygdala and hippocampal subfields, whereas a value nearing 1 signifies an increase in volume. Additionally, violin plots illustrate the z‐scored volumes of the ipsilateral amygdala for further visual interpretation (A). Similarly, the comparison of the volumes of ipsilateral hippocampal subfields between each of the two groups and HCs is also presented, providing a parallel visual and quantitative assessment (B). Further, the comparative analysis of SUVs of ipsilateral and contralateral amygdala and hippocampal HBT subfields for both the HYP‐onset and LVF‐onset groups compared to HCs, quantified using Cohen's d effect size values, with findings visually represented in violin plots (C, D). The violin plots feature orange to denote the relative volumes within the HYP group and red for the LVF group, the ‘*/**/***’ upper the violin indicates the significant results of the ANCOVA analysis (*p* < 0.05/0.01/0.001). AAA, anterior‐amygdala‐area; AB, accessory‐basal nucleus; AMY, amygdala; BA, basal nucleus; Body, hippocampal body; CAT, corticoamygdaloid‐transition; Ce, central nucleus; Co, cortical nucleus; DG‐GC, dentate gyrus granule cells; HATA, hippocampus‐amygdala‐transition‐area; Head, hippocampal head; HIP, hippocampus; LA, lateral nucleus; Me, medial nucleus; PL, paralaminar nucleus; Tail, hippocampal tail.

At the subfield level, the HYP‐onset group showed significantly reduced volumes in all amygdala subfields, and significantly reduced volumes in all hippocampal subfields except HATA and hippocampal fissure. The LVF‐onset group showed significantly increased volumes in the Ba, AB, CAT, and PL and significantly decreased volumes in the Me and no obvious morphological abnormalities were found in any subfield of the hippocampus. The morphological abnormality patterns of the ipsilateral amygdala and hippocampal subfields of patients with MTLE with two SOPs are shown in Figure 2A,B, and detailed statistics are provided in Tables S1–S4.

The hippocampus on the contralateral side in the HYP‐onset group showed no significant changes in volume. Significant atrophy was observed in the Ce and Me of the amygdala. The LVF‐onset group showed significant enlargement in the CAT of the contralateral amygdala and fimbria of the contralateral hippocampus. The morphological abnormality patterns of the contralateral amygdala and hippocampal subfields of patients with MTLE with two SOPs are shown in Figure S2, and detailed statistics are provided in Tables S5–S8.

### 3.2 Metabolic analysis of the amygdala and hippocampal subfields

There was significant ipsilateral hippocampal (HBT subfields) hypometabolism in the HYP‐onset group (HYP *z*‐scores: −2.917 ± 2.847, *p* = 0.013), but not in the LVF‐onset group (LVF *z*‐scores: −0.656 ± 3.510, *p* = 0.795). Neither HYP nor LVF SOPs showed ipsilateral amygdala hypometabolism. At the level of the individual hippocampal subfields, the HYP‐onset group showed significant ipsilateral hypometabolism in the hippocampal head and body. The LVF‐onset group hippocampal metabolism was not significantly different from that of the HCs.

The LVF‐onset group showed significant contralateral hippocampal hypermetabolism (LVF *z*‐scores: 2.111 ± 4.077, *p* = 0.042), which was not observed in the HYP‐onset group (HYP *z*‐scores: 1.438 ± 3.514, *p* = 0.220). At the subfield level, significant hippocampal tail hypermetabolism was observed in both groups of patients, and the LVF‐onset group also exhibited significant contralateral hippocampal body hypermetabolism.

Metabolic abnormalities in the bilateral amygdala and hippocampus are shown in the figure (Figure 2C,D), and detailed statistics are provided in Tables S9–S12.

### 3.3 Pathological analysis of MTLE with both SOPs

**FIGURE 3:**
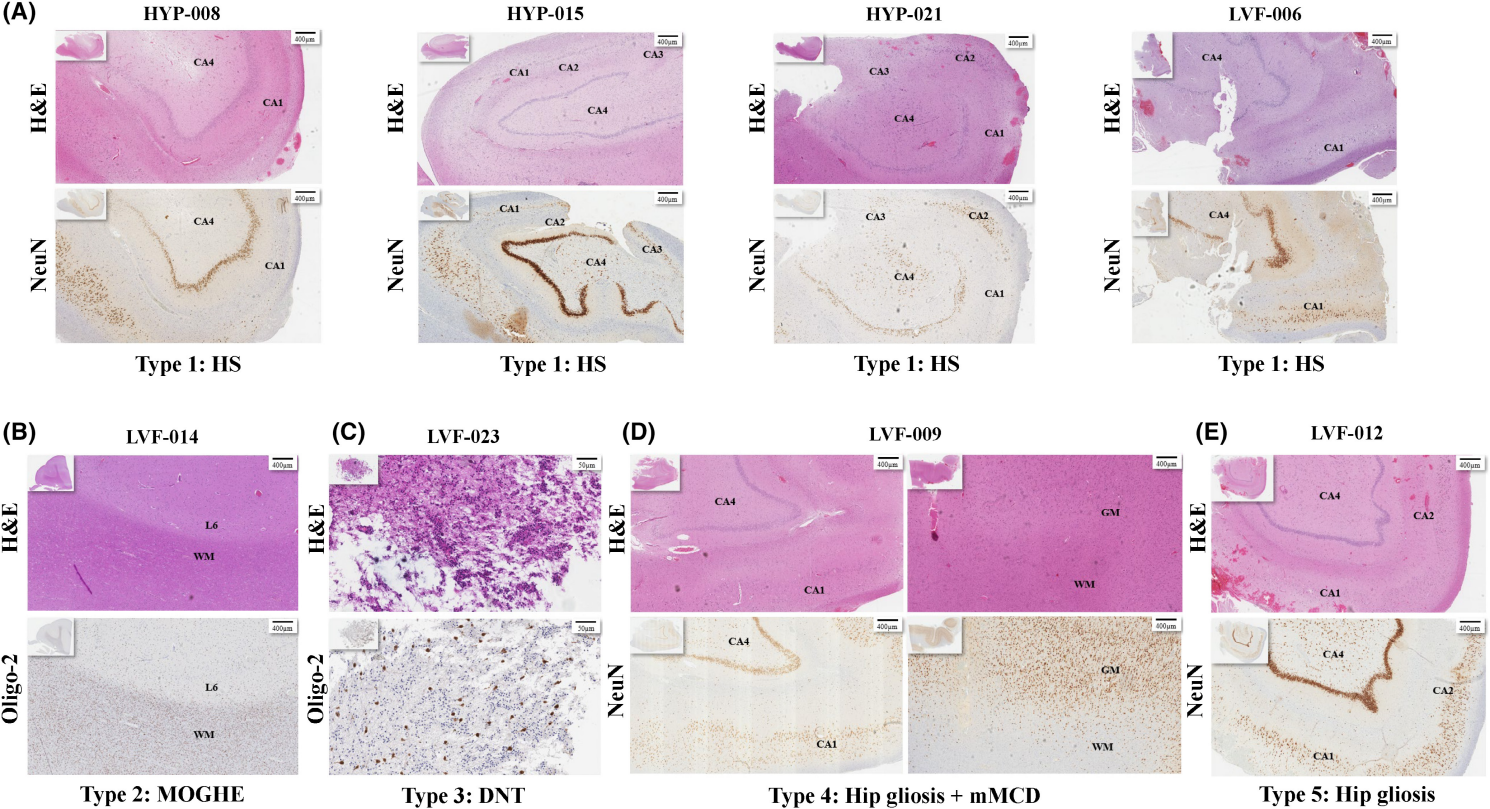
Pathology of MTLE with both SOPs. The pathological results of surgical specimens from 14 MTLE patients who underwent ATLR were mainly divided into the following five types. (A) H&E and NeuN immunohistochemical staining of the resected hippocampus from all 3 cases of HYP‐onset and 1 case of LVF‐onset showed that neuronal loss and gliosis mainly in CA1 and CA4 subfields, meeting the diagnostic criteria for HS. (B) H&E and Oligo‐2 immunohistochemical staining of the resected temporal lobe from 1 case of LVF‐onset showed increased oligodendroglial density in the white matter, meeting the diagnostic criteria for MOGHE. (C) H&E and Oligo‐2 immunohistochemical staining of the resected hippocampal mass lesion from 1 case of LVF‐onset showed typical intracortical nodules having uniform oligodendrocyte‐like cells and floating neurons within microcystic background, meeting the diagnosis of DNT. (D) and (E) respectively demonstrate hippocampal gliosis with or without temporal lobe cortical dysplasia from 2 cases of LVF‐onset group. AAA, anterior‐amygdala‐area; AB, accessory‐basal nucleus; AMY, amygdala; BA, basal nucleus; Body, hippocampal body; CAT, corticoamygdaloid‐transition; Ce, central nucleus; Co, cortical nucleus; DG‐GC, dentate gyrus granule cells; DNT, dysembryoplastic neuroepithelial tumor; HATA, hippocampus‐amygdala‐transition‐area; Head, hippocampal head; HIP, hippocampus; HS, hippocampal sclerosis; LA, lateral nucleus; Me, medial nucleus; mMCD, mild malformation of cortical development; MOGHE, mild malformation of cortical development with oligodendroglial hyperplasia in epilepsy; PL, paralaminar nucleus; Tail, hippocampal tail.

Fourteen patients underwent ATLR surgery: three in the HYP‐onset group and 11 in the LVF‐onset group. All three patients with HYP onset exhibited significant neuronal loss and gliosis in the hippocampus, primarily concentrated in the CA1 and CA4 subfields, meeting the diagnostic criteria for hippocampal sclerosis (HS). In contrast, the destruction of hippocampal structure in patients with LVF onset was less pronounced, and the pathological outcomes were more diverse, mainly including the following five types: HS (1/11), dysembryoplastic neuroepithelial tumor (DNT) (1/11), mild malformation of cortical development with oligodendroglial hyperplasia in epilepsy (MOGHE) (1/11), hippocampal gliosis with mild malformation of cortical development (mMCD) (4/11), and simple hippocampal gliosis (4/11) (Figure 3). These results correspond with the hippocampal morphological and metabolic characteristics of the two groups of patients.

### 3.4 Preoperative diagnosis based on subfield abnormalities

**FIGURE 4:**
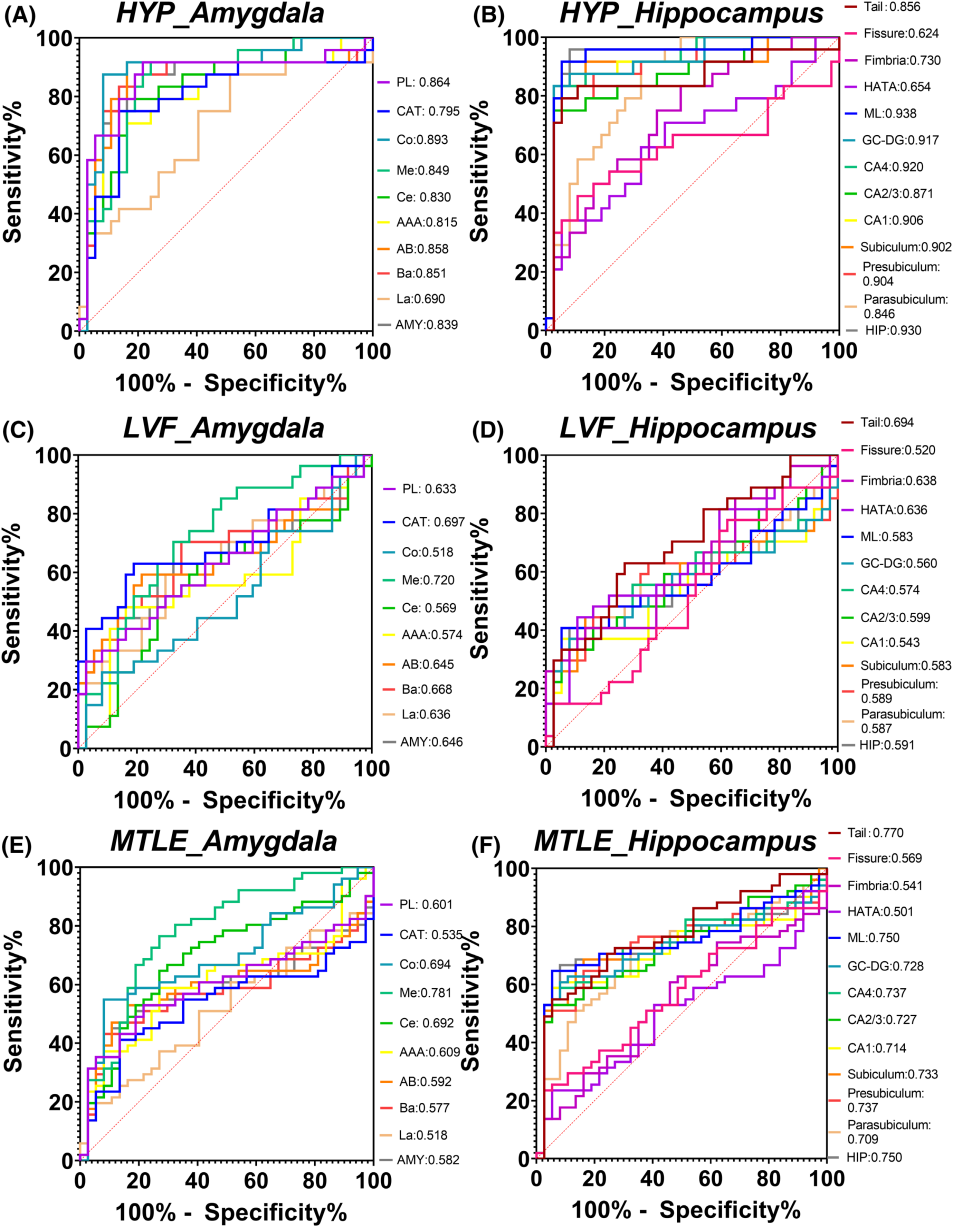
ROC analysis of the ipsilateral amygdala and hippocampal subfield volumes in relation to diagnosis. Classification performance of ipsilateral amygdala and hippocampal subfield volumes in HYP‐onset group (A, B), LVF‐onset group (C, D), and MTLE patients (E, F) are presented by ROC curves and evaluated by AUC. The AUC of the volume of each subfield is shown in the diagram. AAA, anterior‐amygdala‐area; AB, accessory‐basal nucleus; BA, basal nucleus; CAT, corticoamygdaloid‐transition; Ce, central nucleus; Co, cortical nucleus; DG‐GC, dentate gyrus granule cells; HATA, hippocampus‐amygdala‐transition‐area; LA, lateral nucleus; Me, medial nucleus; PL, paralaminar nucleus.

For patients in the HYP group, the best indicator for classification performance was ML volume (area under the curve [AUC] = 0.938), the hippocampus (AUC = 0.930) and other hippocampal subfields, such as CA1 (AUC = 0.906), CA4 (AUC = 0.920), GC‐DG (AUC = 0.917), and presubiculum (AUC = 0.904), exhibited similar classification performance (Figure 4A,B). For the LVF group, the classification performance of the amygdala and hippocampal subfield volume was lower than that of the HYP group. The best indicator was Me volume (AUC = 0.720), which was better than the classification performance of the amygdala (AUC = 0.646) and the hippocampus (AUC = 0.591) and its subfields (Figure 4C,D).

The two groups of MTLE patients with different SOPs were mixed to evaluate the classification performance of the amygdala and hippocampal subfield volume in the preoperative diagnosis of patients with MTLE. Me volume was the best indicator (AUC = 0.781), exceeding the whole amygdala (AUC = 0.582) and hippocampus (AUC = 0.750) (Figure 4E,F).

For the HYP group, hypometabolism of the amygdala (AUC = 0.817), hippocampus (AUC = 0.830), and hippocampal head and body had ideal classification performance, and the best indicator was hypometabolism of the hippocampal head (AUC = 0.872) (Figure S3A). For the LVF group, compared with hypometabolism of the hippocampus (AUC = 0.556) and its HBT subfields, hypometabolism of the amygdala (AUC = 0.704) had better classification performance, although their classification performances were lower than those of the HYP group (Figure S3B). On mixing the two groups of patients with MTLE with different SOPs to evaluate the performance of metabolic abnormalities in the amygdala and hippocampal HBT subfields in preoperative MTLE diagnosis, the better indicators were hypometabolism of the amygdala (AUC = 0.758) and hippocampal head (AUC = 0.750), which were better than that of the hippocampus (AUC = 0.703) (Figure S3C).

### 3.5 Prognosis prediction based on subfield abnormalities

Compared with the LVF‐onset group, the HYP‐onset group had a trend of better prognosis. 71.4% of the HYP‐onset group (15/21) receiving RFTC treatment became seizure‐free (ILAE class 1), while the LVF‐onset group only had 43.8% (7/16); 66.6% of patients (2/3) receiving ATLR in the HYP‐onset group achieved ILAE class 1, while only 45.5% (5/11) in the LVF‐onset group. With the small sample size, no significant differences were found.

**FIGURE 5:**
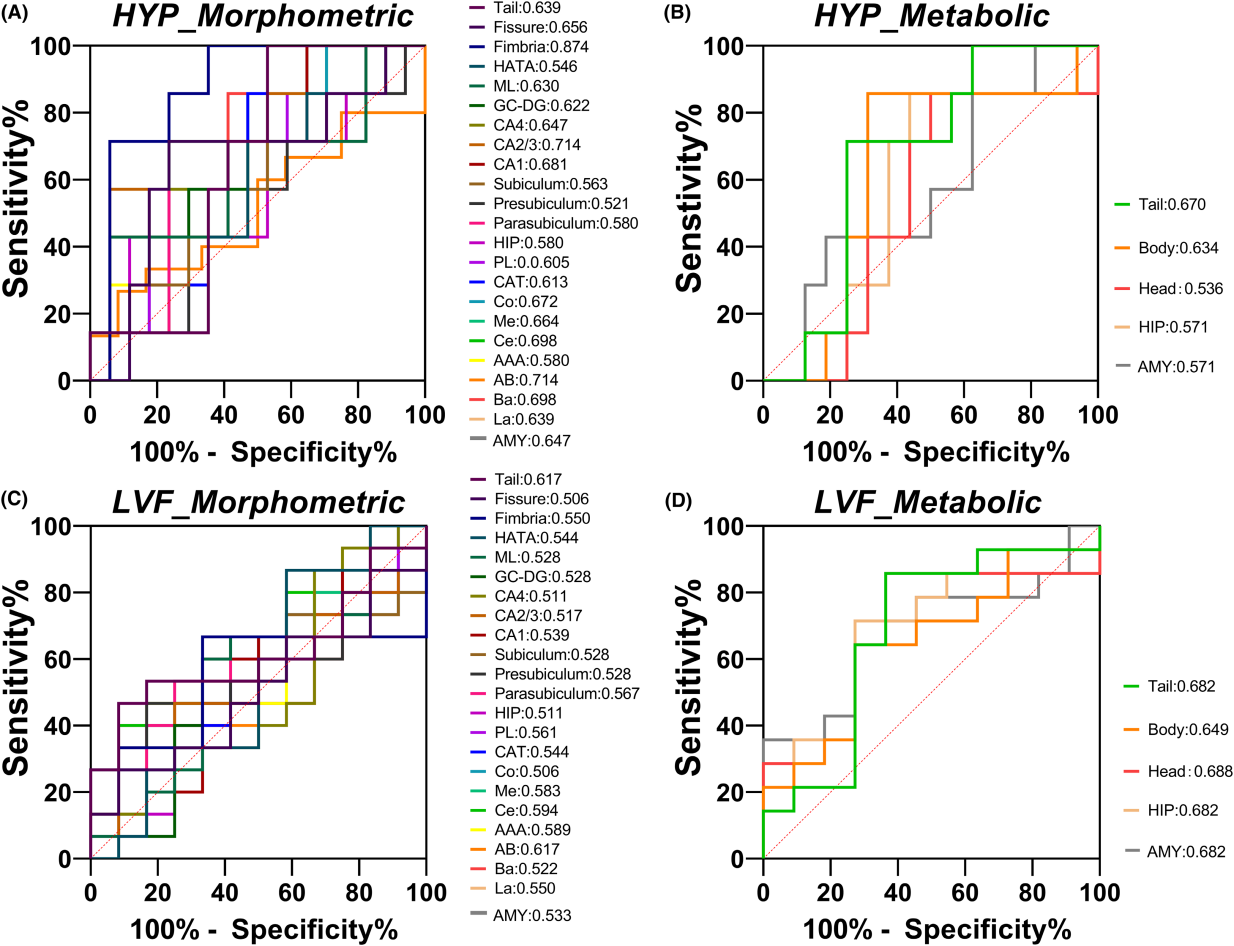
ROC analysis of the ipsilateral amygdala and hippocampal subfield abnormalities in relation to prognosis. Prognosis prediction performance of ipsilateral amygdala and hippocampal subfield volumes in HYP‐onset group and LVF‐onset group (A, C), and those of ipsilateral amygdala and hippocampal HBT subfield in two groups (B, D), are presented by ROC curves and evaluated by AUC. The AUC of the volume and SUV of each subfield is shown in the diagram. AAA, anterior‐amygdala‐area; AB, accessory‐basal nucleus; AMY, amygdala; BA, basal nucleus; Body, hippocampal body; CAT, corticoamygdaloid‐transition; Ce, central nucleus; Co, cortical nucleus; DG‐GC, dentate gyrus granule cells; HATA, hippocampus‐amygdala‐transition‐area; Head, hippocampal head; HIP, hippocampus; LA, lateral nucleus; Me, medial nucleus; PL, paralaminar nucleus; Tail, hippocampal tail.

In the HYP‐onset group, atrophy of the fimbria was associated with unsatisfactory surgical prognosis (AUC = 0.874) (Figure 5A), which is better than that of the amygdala (AUC = 0.647) and hippocampus (AUC = 0.580). Hypometabolism in the amygdala (AUC = 0.571) and hippocampus (AUC = 0.571), along with its HBT subfields, did not enable prediction of prognosis (Figure 5B). For the LVF‐onset group, no indicators with high predictive value for prognosis were found (Figure 5C,D).

## 4 DISCUSSION

Few studies have simultaneously explored the morphological and metabolic abnormalities in the amygdala and hippocampal subfields of MTLE patients with different SOPs.^13^, ^22^, ^23^, ^24^ The main findings of this study were:

1. Patients with HYP‐onset had atrophy and hypometabolism in the ipsilateral amygdala, hippocampus, including their subfields, which showed excellent classification performance in diagnosing this subtype of MTLE. These features demonstrated superior diagnostic accuracy for this MTLE subtype. Pathologically, these cases were confirmed to have HS. Furthermore, pronounced atrophy of the ipsilateral fimbria was linked to an unfavorable prognosis following surgery.
2. Patients with LVF‐onset MTLE only exhibited ipsilateral amygdala enlargement (AE). At the subfield level, enlargement of the Ba, AB, CAT, PL, and Me atrophy were observed. No morphological or metabolic abnormalities were observed in the ipsilateral hippocampal subfields, pathological verification also supports this characteristic. Me atrophy was the most reliable indicator for diagnosing patients with LVF‐onset MTLE, but no indicator effectively predicted surgical prognosis.
3. There was a significant difference between the two SOPs: patients in the HYP‐onset group had more definite hippocampal structural and metabolic abnormalities, whereas the LVF‐onset group had abnormalities mainly reflected in the amygdala rather than the hippocampus, and the pathological results were more diverse, the seizures were more frequent, and the surgical prognosis was relatively poor.
4. MTLE patients often have abnormalities in the contralateral amygdala and hippocampus. For the HYP‐onset group, there was atrophy of Me and Ce in the contralateral amygdala. For the LVF‐onset group, there was hypermetabolism in the contralateral hippocampus. Both groups of patients had hypermetabolism in the contralateral hippocampal tail.

In patients with HYP‐onset MTLE, we found typical atrophy in multiple subfields of the ipsilateral hippocampus. Considering previous reports that HA is often accompanied by HS, and the pathological basis for patients in the HYP‐onset group who underwent surgical resection in this study was uniformly HS, we believe that HYP onset is highly correlated with HS.^3^, ^9^, ^25^ Other imaging features of HS, such as amygdala atrophy^22^, ^26^ and hippocampal hypometabolism concentrated in the head and body, were also exhibited in the HYP group, which further confirmed our view. This result is consistent with the research results of Lam et al.,^27^ although we are referring specifically to HYP‐onset MTLE.

Schönberger et al. found that the mechanisms underlying HYP‐onset seizures and fast ripple (250–500 Hz) are caused by abnormal excitation of pyramidal cells (glutamatergic) in the hippocampus.^25^, ^28^ Engel and Bragin found that HYP‐onset seizures were limited to the medial temporal lobe and rarely propagated extratemporal lobe by SEEG.^15^ Based on the above research results and our findings, we suggest that frequent and focal seizures in the medial temporal lobe can lead to hippocampal pyramidal cell damage due to glutamatergic excitotoxicity,^27^, ^29^ causing structural and metabolic abnormalities manifesting as HA and hypometabolism of the hippocampal head and body,

Patients with HYP‐onset MTLE have a good surgical prognosis.^15^ In this study, patients with HYP‐onset MTLE had an overall seizure‐free rate of 70.8%, with a seizure‐free rate of 71.4% (15/21) in patients receiving RFTC and 66.7% (2/3) in patients receiving ATLR. However, for patients who did not become seizure‐free after surgery, we observed severe fimbria atrophy, which plays an important role in the Papez circuit.^30^, ^31^ This may indicate that HYP‐onset patients with MTLE who do not become seizure‐free are more likely to involve the limbic system through the Papez circuit,^32^ and the surgical outcome will depend on whether the fimbria and connections to the fornix are resected or ablated. Oikawa et al. reported that abnormalities in the Papez circuit are potential factors for poor prognosis in patients with MTLE.^30^ Therefore, a comprehensive SEEG evaluation of the limbic system and expanding the surgical resection or ablation towards the posterior medial side of the hippocampus for patients with HYP‐onset MTLE and severe fimbria atrophy may be an appropriate consideration.

In contrary to the HYP‐onset group, none of the hippocampal subfields in patients with LVF‐onset MTLE showed any structural or metabolic abnormalities despite SEEG confirming that the seizures originated from the medial temporal lobe. Velasco reported that the loss of hippocampal neurones in patients with LVF‐onset MTLE was significantly less than that in patients with HYP‐onset MTLE. Only 42.9% of patients with LVF‐onset MTLE were diagnosed with HS, whereas all patients with HYP‐onset MTLE were diagnosed with HS.^9^ In the patient cohort of this study, among 11 patients, only one was diagnosed with HS, and most other pathological diagnoses were hippocampal gliosis with or without temporal cortex abnormalities. Thus, patients with LVF‐onset MTLE are usually MRI‐negative. The explanation may be that the LVF‐onset MTLE is mainly related to the abnormal excitation of hippocampal interneurones (GABAergic), which subsequently causes excitement of hippocampal pyramidal cells (glutamatergic),^25^, ^33^ and the epileptic activity will spread rapidly from the hippocampus to the extratemporal or contralateral side of the brain,^2^, ^5^, ^13^ causing less damage to the hippocampal pyramidal cells.^9^ In consequence, finding in vivo diagnostic imaging evidence for MTLE with LVF‐onset SOP is difficult.

For the first time, we confirmed the relationship between AE and LVF‐onset MTLE. Although numerous studies have demonstrated that AE is a unique MTLE subtype, recent research has found it to be a morphological biomarker in patients with MRI‐negative MTLE.^34^, ^35^, ^36^ We found that the Ba, AB, and PL subfields of the amygdala were significantly enlarged. These subfields project to the prefrontal cortex, insula, sensory cortex, and ventral hippocampus via extensive afferent and efferent circuits.^37^, ^38^ It is suggested that MTLE with AE involves a wider epileptogenic network, including temporal‐insular and temporal‐prefrontal types,^39^ which is consistent with the characteristics of LVF‐onset MTLE,^5^, ^9^ and resulting in a less good surgical prognosis than in those with HYP‐onset MTLE.^39^ However, the quantitative classification of amygdala abnormalities is not ideal, and AE cannot be the basis for diagnosing LVF‐onset MTLE. There were also no ideal indicators in predicting surgical prognosis in the LVF‐onset group; therefore, accurate evaluation of the epileptogenic network through individualized SEEG implantation is crucial for achieving better prognosis in patients with MTLE without hippocampal atrophy.

Overall, comparing MTLE patients with different SOPs, we found that patients in the HYP‐onset group had more well‐defined hippocampal structural and metabolic abnormalities, and the pathological results also supported this finding, suggesting that it was highly correlated with HS. In contrast, compared with hippocampal abnormalities, AE was a more specific imaging abnormality in patients in the LVF‐onset group, and pathological results also suggested that patients in this group usually did not have serious hippocampal abnormalities, usually simple gliosis with or without temporal neocortex and white matter abnormalities. Thus, HYP onset suggests a more limited epilepsy network, while LVF onset may represent a more complex epilepsy network, which is supported by the clinical characteristics of both groups, with the LVF‐onset group typically having more frequent seizures and a slightly worse surgical prognosis than those in the HYP‐onset group.

There are similarities between the two groups of patients. The Me, as a component of the central‐medial complex of the amygdala,^26^ showed significant atrophy in patients with MTLE with both SOPs. Owing to the extensive atrophy of the medial temporal lobe structure in patients with HYP‐onset MTLE, Me atrophy is not unexpected. However, in the context of overall AE, isolated atrophy of the Me in patients with LVF‐onset MTLE is interesting, as the Me mainly receives projections from the olfactory bulb, Further research into the pathophysiological role of Me would be of interest.

Patients with MTLE of both SOPs exhibit varying degrees of hypermetabolism of the contralateral hippocampus. In the HYP‐onset group, this was mainly concentrated in the hippocampal tail, while this involved the body and tail of the hippocampus in the LVF‐onset group, and the metabolism of the entire hippocampus was significantly increased, possibly due to compensation for ipsilateral hippocampal function.

This study had some limitations. First, the reproducibility of the results needs to be investigated further with larger samples at other centers. Second, the ^18^F‐FDG‐PET data of HCs used in this study were obtained from the OpenNeuro Dataset. Although SUVR and z‐score transformation were performed, image data from different institutions may still have influenced the results. Third, when comparing the prognosis of patients with MTLE of both SOPs, different surgical methods may have influenced the results.

## 5 CONCLUSION

In conclusion, this study identified morphological and metabolic abnormalities of the amygdala and hippocampal subfields in patients with MTLE with two different SOPs and clarified the value of these abnormalities for clinical diagnosis and prognosis.

## AUTHOR CONTRIBUTIONS

T.F., Y.Y. and Y.W. contributed to the conception and design of the study. T.F., Y.Y. contributed to data collection. T.F., Y.W., H.Z., Y.A., T.W. and Y.H. contributed to analysis of data. T.F., J.S.D., P.W., X.F., S.C. and F.X. contributed to drafting the text, preparing the figures. Y.S. and G.Z. operated patients and interpreting the results. All authors reviewed and revised the manuscript for intellectual content.

## CONFLICT OF INTEREST STATEMENT

The authors declare that they have no conflict of interest. We confirm that we have read the Journal's position on issues involved in ethical publication and affirm that this report is consistent with those guidelines.

## PATIENT CONSENT STATEMENT

All participants provided written informed consent.

## PERMISSION TO REPRODUCE MATERIAL FROM OTHER SOURCES

None.

## Supporting information


Data S1

## Data Availability

Statistical data for replicating key findings can be requested from the corresponding author by qualified investigators. Other data are restricted to protect participant privacy.
