# Supplementary material for: Delineating structural and metabolic abnormalities in amygdala and hippocampal subfields for different seizure‐onset patterns via stereotactic electroencephalography
Source: CNS Neurosci Ther. 2024 Sep 9;30(9):e14905. doi: 10.1111/cns.14905 (PMC11382356; doi:10.1111/cns.14905)
Supplement: Supplementary file 1 — Data S1 [file CNS-30-e14905-s001.zip › Supplementary Figure.docx]

**Supplementary Fig.1 Amygdala and hippocampal HBT subfields segmentation of structural MRI and ^18^F-FDG-PET.** Preoperative structural MRI to segment the amygdala and the hippocampal subfields, PET and MRI were co-registered, then used the masks segmented by structural MRI to extract the SUV. SUV: standard uptake value.


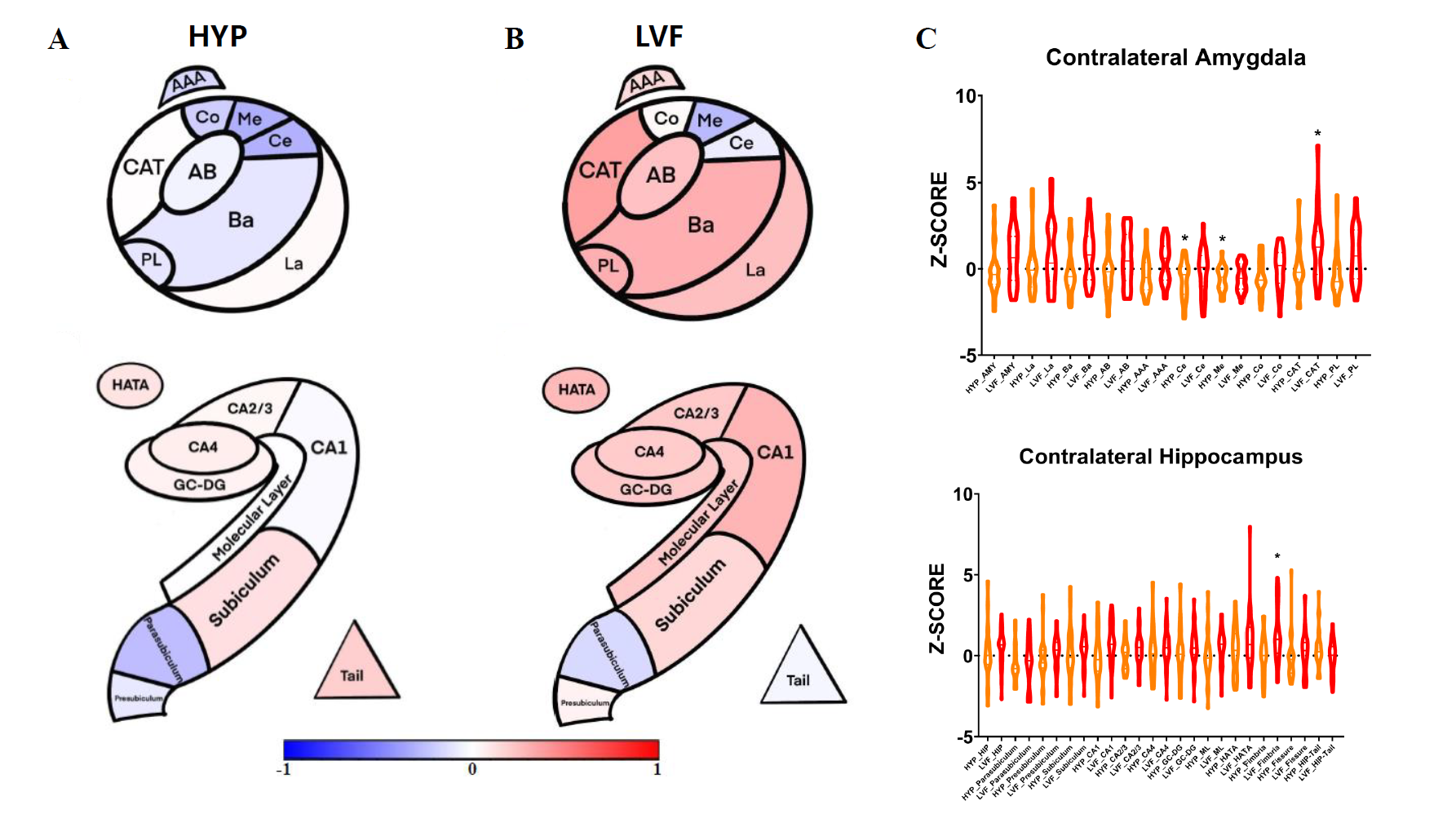


**Supplementary Fig.2 Volumetric analysis of the contralateral amygdala and hippocampal subfields between patients and HCs.** Graphic representations and violin plots of the contralateral amygdala and hippocampal subfields volumes’ comparisons between patients and HCs is presented. The comparison between HYP-onset group and HCs (A), and LVF-onset group and HCs (B) are represented with Cohens’ d effect size value, a value close to -1 reflects the decrease in the amygdala and hippocampal subfields volume, while a value near 1 refers to the increase in volume. Violine plots of z-scored volumes of the contralateral amygdala (C) and hippocampal subfields (D) in HYP-onset group and LVF-onset group patients relative to HCs, a line of 0 represents the mean volume of HCs. The orange represents the relative volume of the HYP group, and the red represents that of the LVF group, the ‘*/**/***’ upper the violine indicates the significant results of the ANCOVA analysis (P < 0.05/0.01/0.001). LA: lateral nucleus, BA: basal nucleus, AB: accessory-basal nucleus, AAA: anterior-amygdala-area, Ce: central nucleus, Me: medial nucleus, Co: cortical nucleus, CAT: corticoamygdaloid-transitio, PL: paralaminar nucleus, DG-GC: dentate gyrus granule cells, HATA: hippocampus-amygdala-transition-area.


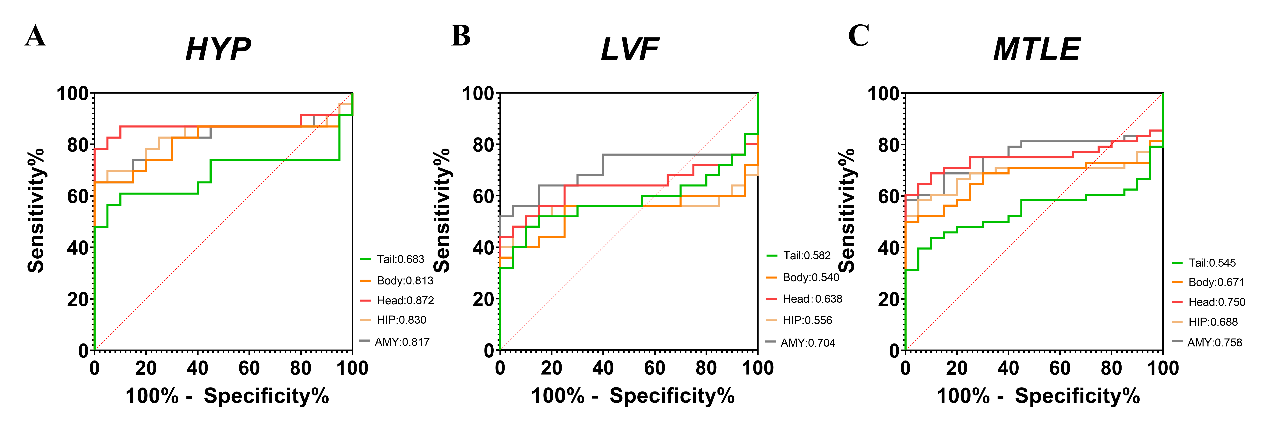


**Supplementary Fig.3 ROC analysis of the ipsilateral amygdala and hippocampal HBT subfield SUVs in relation to diagnosis.** Classification performance of ipsilateral amygdala and hippocampal HBT subfield SUVs in HYP-onset group with HCs (A), LVF-onset group with HCs (B), and MTLE patients with HCs (C) are presented by ROC curves and evaluated by AUC. The AUC of the SUV of each subfield is shown in the diagram. AMY: amygdala, HIP: hippocampus, Head: hippocampal head, Body: hippocampal body, Tail: hippocampal tail.
