# Supplementary material for: Delineating structural and metabolic abnormalities in amygdala and hippocampal subfields for different seizure‐onset patterns via stereotactic electroencephalography
Source: CNS Neurosci Ther. 2024 Sep 9;30(9):e14905. doi: 10.1111/cns.14905 (PMC11382356; doi:10.1111/cns.14905)
Supplement: Supplementary file 1 — Data S1 [file CNS-30-e14905-s001.zip › Supplementary materials-Table.docx]

Supplementary Table 1: Morphometric comparison of ipsilateral amygdala subfields between MTLE patients and HC

|  | HYP _z-score_ | LVF _z-score_ | P-value _(HYP-HC)_ | P-value _(LVF-HC)_ | t _(HYP-HC)_ | t _(LVF-HC)_ | Cohen’s d _(HYP-HC)_ | Cohen’s d _(LVF-HC)_ |
| --- | --- | --- | --- | --- | --- | --- | --- | --- |
| Whole-amy | -1.182±1.427 | 0.722±1.540 | <0.001* | 0.048* | -3.552 | 2.323 | -0.432 | 0.268 |
| La | -0.760±1.768 | 0.769±1.850 | 0.043* | 0.060 | -2.054 | 2.064 | -0.256 | 0.250 |
| Ba | -1.280±1.334 | 0.762±1.434 | <0.001* | 0.037* | -4.008 | 2.599 | -0.477 | 0.295 |
| AB | -1.191±1.187 | 0.617±1.286 | <0.001* | 0.048* | -4.015 | 2.280 | -0.477 | 0.259 |
| AAA | -1.214±1.094 | 0.306±1.173 | <0.001* | 0.233 | -4.202 | 1.332 | -0.501 | 0.139 |
| Ce | -1.049±0.852 | -0.069±0.945 | <0.001* | 0.888 | -4.269 | -0.141 | -0.492 | -0.035 |
| Me | -1.056±0.584 | -0.630±0.699 | <0.001* | 0.020* | -5.035 | -2.966 | -0.542 | -0.343 |
| Co | -1.243±0.784 | -0.062±0.863 | <0.001* | 0.888 | -5.359 | -0.188 | -0.569 | -0.033 |
| CAT | -1.022±1.245 | 1.121±1.765 | 0.004* | 0.007* | -2.990 | 3.494 | -0.412 | 0.364 |
| PL | -1.496±1.414 | 0.739±1.635 | <0.001* | 0.048* | -4.336 | 2.224 | -0.521 | 0.263 |

Supplementary Table 2: Table of between-subjects effects for ANCOVA analysis of ipsilateral amygdala subfields volume

| Source | Structural | df | F | P |
| --- | --- | --- | --- | --- |
| Corrected Model | whole_amy | 4 | 7.430 | <0.001 |
|  | La | 4 | 3.633 | 0.009 |
|  | Ba | 4 | 9.425 | <0.001 |
|  | AB | 4 | 8.664 | <0.001 |
|  | AAA | 4 | 7.545 | <0.001 |
|  | Ce | 4 | 5.465 | 0.001 |
|  | Me | 4 | 6.723 | <0.001 |
|  | Co | 4 | 8.385 | <0.001 |
|  | CAT | 4 | 8.931 | <0.001 |
|  | PL | 4 | 9.416 | <0.001 |
| Intercept | whole_amy | 1 | 0.203 | 0.653 |
|  | La | 1 | 0.076 | 0.783 |
|  | Ba | 1 | 0.384 | 0.537 |
|  | AB | 1 | 0.254 | 0.616 |
|  | AAA | 1 | 0.513 | 0.476 |
|  | Ce | 1 | 0.007 | 0.932 |
|  | Me | 1 | 1.661 | 0.201 |
|  | Co | 1 | 0.489 | 0.487 |
|  | CAT | 1 | 1.075 | 0.303 |
|  | PL | 1 | 0.016 | 0.899 |
| Sex | whole_amy | 1 | 2.131 | 0.148 |
|  | La | 1 | 1.466 | 0.229 |
|  | Ba | 1 | 2.409 | 0.124 |
|  | AB | 1 | 2.056 | 0.155 |
|  | AAA | 1 | 1.626 | 0.206 |
|  | Ce | 1 | 1.525 | 0.220 |
|  | Me | 1 | 0.811 | 0.370 |
|  | Co | 1 | 1.441 | 0.233 |
|  | CAT | 1 | 2.765 | 0.100 |
|  | PL | 1 | 1.003 | 0.319 |
| Age | whole_amy | 1 | 0.051 | 0.821 |
|  | La | 1 | 0.271 | 0.604 |
|  | Ba | 1 | 0.001 | 0.971 |
|  | AB | 1 | 0.001 | 0.971 |
|  | AAA | 1 | 0.335 | 0.565 |
|  | Ce | 1 | 0.021 | 0.884 |
|  | Me | 1 | 0.605 | 0.439 |
|  | Co | 1 | 0.485 | 0.488 |
|  | CAT | 1 | 0.003 | 0.958 |
|  | PL | 1 | 0.219 | 0.641 |
| Groups | whole_amy | 2 | 14.727 | <0.001 |
|  | La | 2 | 7.102 | <0.001 |
|  | Ba | 2 | 18.638 | <0.001 |
|  | AB | 2 | 17.133 | <0.001 |
|  | AAA | 2 | 14.295 | <0.001 |
|  | Ce | 2 | 10.600 | <0.001 |
|  | Me | 2 | 13.199 | <0.001 |
|  | Co | 2 | 16.678 | <0.001 |
|  | CAT | 2 | 17.652 | <0.001 |
|  | PL | 2 | 18.818 | <0.001 |
| Error | whole_amy | 83 | - | - |
|  | La | 83 | - | - |
|  | Ba | 83 | - | - |
|  | AB | 83 | - | - |
|  | AAA | 83 | - | - |
|  | Ce | 83 | - | - |
|  | Me | 83 | - | - |
|  | Co | 83 | - | - |
|  | CAT | 83 | - | - |
|  | PL | 83 | - | - |
| Total | whole_amy | 88 | - | - |
|  | La | 88 | - | - |
|  | Ba | 88 | - | - |
|  | AB | 88 | - | - |
|  | AAA | 88 | - | - |
|  | Ce | 88 | - | - |
|  | Me | 88 | - | - |
|  | Co | 88 | - | - |
|  | CAT | 88 | - | - |
|  | PL | 88 | - | - |
| Corrected Total | whole_amy | 87 | - | - |
|  | La | 87 | - | - |
|  | Ba | 87 | - | - |
|  | AB | 87 | - | - |
|  | AAA | 87 | - | - |
|  | Ce | 87 | - | - |
|  | Me | 87 | - | - |
|  | Co | 87 | - | - |
|  | CAT | 87 | - | - |
|  | PL | 87 | - | - |

Supplementary Table 3: Morphometric comparison of ipsilateral hippocampal subfields between MTLE patients and HC

|  | HYP _z-score_ | LVF _z-score_ | P-value _(HYP-HC)_ | P-value _(LVF-HC)_ | t _(HYP-HC)_ | t _(LVF-HC)_ | Cohen’s d _(HYP-HC)_ | Cohen’s d _(LVF-HC)_ |
| --- | --- | --- | --- | --- | --- | --- | --- | --- |
| Whole-hip | -2.300±1.211 | -0.388±1.361 | <0.001* | 0.552 | -7.508 | -1.120 | -0.719 | -0.160 |
| Parasubiculum | -1.313±0.949 | -0.332±1.614 | <0.001* | 0.552 | -3.870 | -0.863 | -0.559 | -0.123 |
| Presubiculum | -1.900±1.027 | -0.273±1.181 | <0.001* | 0.552 | -6.063 | -0.885 | -0.684 | -0.124 |
| Subiculum | -2.115±1.255 | -0.371±1.310 | <0.001* | 0.552 | -7.332 | -1.098 | -0.682 | -0.157 |
| CA1 | -2.145±1.221 | -0.125±1.632 | <0.001* | 0.765 | -6.423 | -0.300 | -0.693 | 0.046 |
| CA2/3 | -1.510±1.012 | -0.275±1.208 | <0.001* | 0.552 | -50253 | -0.771 | -0.600 | -0.123 |
| CA4 | -2.255±1.256 | -0.319±1.324 | <0.001* | 0.552 | -7.163 | -0.829 | -0.705 | -0.135 |
| GC-DG | -2.233±1.235 | -0.286±1.319 | <0.001* | 0.552 | -7.166 | -0.730 | -0.705 | -0.121 |
| ML | -2.335±1.147 | -0.398±1.364 | <0.001* | 0.552 | -7.629 | -1.145 | -0.719 | -0.164 |
| HATA | -0.518±1.092 | 0.785±1.746 | 0.111 | 0.052 | -1.611 | 2.715 | -0.240 | 0.266 |
| Fimbria | -0.748±0.817 | 0.878±1.737 | 0.021* | 0.056 | -2.403 | 2.532 | -0.379 | 0.296 |
| Hip-fissure | -0.414±1.603 | 0.122±1.780 | 0.111 | 0.726 | -1.654 | 0.427 | -0.153 | 0.042 |
| Hip-tail | -1.455±1.242 | -0.740±1.016 | <0.001* | 0.052 | -5.526 | -2.710 | -0.542 | -0.345 |

Supplementary Table 4: Table of between-subjects effects for ANCOVA analysis of ipsilateral hippocampal subfields volume

| Source | Structural | df | F | P |
| --- | --- | --- | --- | --- |
| Corrected Model | Whole_hip | 4 | 15.484 | <0.001 |
|  | Parasubiculum | 4 | 4.861 | 0.001 |
|  | Presubiculum | 4 | 10.455 | <0.001 |
|  | Subiculum | 4 | 14.962 | <0.001 |
|  | CA1 | 4 | 11.944 | <0.001 |
|  | CA2/3 | 4 | 8.114 | <0.001 |
|  | CA4 | 4 | 14.895 | <0.001 |
|  | GC-DG | 4 | 14.943 | <0.001 |
|  | ML | 4 | 16.153 | <0.001 |
|  | HATA | 4 | 4.564 | 0.002 |
|  | Fimbria | 4 | 6.051 | <0.001 |
|  | Hip_fissure | 4 | 2.864 | 0.028 |
|  | Hip_tail | 4 | 8.078 | <0.001 |
| Intercept | Whole_hip | 1 | 0.458 | 0.500 |
|  | Parasubiculum | 1 | 0.281 | 0.597 |
|  | Presubiculum | 1 | 0.000 | 0.994 |
|  | Subiculum | 1 | 0.768 | 0.383 |
|  | CA1 | 1 | 0.703 | 0.404 |
|  | CA2/3 | 1 | 0.072 | 0.789 |
|  | CA4 | 1 | 0.006 | 0.938 |
|  | GC-DG | 1 | 0.011 | 0.915 |
|  | ML | 1 | 0.248 | 0.620 |
|  | HATA | 1 | 2.762 | 0.100 |
|  | Fimbria | 1 | 1.789 | 0.185 |
|  | Hip_fissure | 1 | 0.146 | 0.703 |
|  | Hip_tail | 1 | 2.748 | 0.101 |
| sex | Whole_hip | 1 | 2.402 | 0.125 |
|  | Parasubiculum | 1 | 0.223 | 0.638 |
|  | Presubiculum | 1 | 2.699 | 0.104 |
|  | Subiculum | 1 | 5.429 | 0.022 |
|  | CA1 | 1 | 1.117 | 0.294 |
|  | CA2/3 | 1 | 1.461 | 0.230 |
|  | CA4 | 1 | 1.574 | 0.213 |
|  | GC-DG | 1 | 1.729 | 0.192 |
|  | ML | 1 | 2.002 | 0.161 |
|  | HATA | 1 | 4.415 | 0.039 |
|  | Fimbria | 1 | 0.675 | 0.414 |
|  | Hip_fissure | 1 | 5.526 | 0.021 |
|  | Hip_tail | 1 | 1.984 | 0.163 |
| age | Whole_hip | 1 | 0.101 | 0.752 |
|  | Parasubiculum | 1 | 1.651 | 0.202 |
|  | Presubiculum | 1 | 0.024 | 0.876 |
|  | Subiculum | 1 | 1.429 | 0.235 |
|  | CA1 | 1 | 0.290 | 0.592 |
|  | CA2/3 | 1 | 0.429 | 0.514 |
|  | CA4 | 1 | 0.315 | 0.576 |
|  | GC-DG | 1 | 0.211 | 0.647 |
|  | ML | 1 | 0.002 | 0.963 |
|  | HATA | 1 | 0.177 | 0.675 |
|  | Fimbria | 1 | 1.322 | 0.254 |
|  | Hip_fissure | 1 | 3.968 | 0.050 |
|  | Hip_tail | 1 | 2.521 | 0.116 |
| groups | Whole_hip | 2 | 30.350 | <0.001 |
|  | Parasubiculum | 2 | 7.776 | 0.001 |
|  | Presubiculum | 2 | 19.831 | <0.001 |
|  | Subiculum | 2 | 28.940 | <0.001 |
|  | CA1 | 2 | 23.755 | <0.001 |
|  | CA2/3 | 2 | 14.877 | <0.001 |
|  | CA4 | 2 | 28.185 | <0.001 |
|  | GC-DG | 2 | 28.453 | <0.001 |
|  | ML | 2 | 31.320 | <0.001 |
|  | HATA | 2 | 8.054 | 0.001 |
|  | Fimbria | 2 | 10.201 | <0.001 |
|  | Hip_fissure | 2 | 2.083 | 0.131 |
|  | Hip_tail | 2 | 15.409 | <0.001 |
| Error | Whole_hip | 83 | - | - |
|  | Parasubiculum | 83 | - | - |
|  | Presubiculum | 83 | - | - |
|  | Subiculum | 83 | - | - |
|  | CA1 | 83 | - | - |
|  | CA2/3 | 83 | - | - |
|  | CA4 | 83 | - | - |
|  | GC-DG | 83 | - | - |
|  | ML | 83 | - | - |
|  | HATA | 83 | - | - |
|  | Fimbria | 83 | - | - |
|  | Hip_fissure | 83 | - | - |
|  | Hip_tail | 83 | - | - |
| Total | Whole_hip | 88 | - | - |
|  | Parasubiculum | 88 | - | - |
|  | Presubiculum | 88 | - | - |
|  | Subiculum | 88 | - | - |
|  | CA1 | 88 | - | - |
|  | CA2/3 | 88 | - | - |
|  | CA4 | 88 | - | - |
|  | GC-DG | 88 | - | - |
|  | ML | 88 | - | - |
|  | HATA | 88 | - | - |
|  | Fimbria | 88 | - | - |
|  | Hip_fissure | 88 | - | - |
|  | Hip_tail | 88 | - | - |
| Corrected Total | Whole_hip | 87 | - | - |
|  | Parasubiculum | 87 | - | - |
|  | Presubiculum | 87 | - | - |
|  | Subiculum | 87 | - | - |
|  | CA1 | 87 | - | - |
|  | CA2/3 | 87 | - | - |
|  | CA4 | 87 | - | - |
|  | GC-DG | 87 | - | - |
|  | ML | 87 | - | - |
|  | HATA | 87 | - | - |
|  | Fimbria | 87 | - | - |
|  | Hip_fissure | 87 | - | - |
|  | Hip_tail | 87 | - | - |

Supplementary Table 5: Morphometric comparison of contralateral amygdala subfields between MTLE patients and HC

|  | HYP _z-score_ | LVF _z-score_ | P-value _(HYP-HC)_ | P-value _(LVF-HC)_ | t _(HYP-HC)_ | t _(LVF-HC)_ | Cohen’s d _(HYP-HC)_ | Cohen’s d _(LVF-HC)_ |
| --- | --- | --- | --- | --- | --- | --- | --- | --- |
| Whole-amy | -0.147±1.402 | 0.742±1.614 | 0.388 | 0.084 | -1.075 | 2.070 | -0.060 | 0.266 |
| La | 0.063±1.550 | 0.762±1.873 | 0.673 | 0.100 | -0.518 | 1.904 | 0.024 | 0.246 |
| Ba | -0.229±1.307 | 0.813±1.515 | 0.323 | 0.063 | -1.310 | 2.391 | -0.098 | 0.302 |
| AB | -0.153±1.360 | 0.600±1.497 | 0.388 | 0.127 | -1.021 | 1.723 | -0.064 | 0.229 |
| AAA | -0.356±1.105 | 0.343±1.180 | 0.208 | 0.311 | -1.754 | 1.153 | -0.167 | 0.155 |
| Ce | -0.656±1.066 | -0.173±1.278 | 0.040* | 0.311 | -2.920 | -1.087 | -0.302 | -0.075 |
| Me | -0.570±0.717 | -0.502±0.770 | 0.040* | 0.060 | -2.735 | -2.555 | -0.311 | -0.271 |
| Co | -0.396±0.980 | 0.045±1.147 | 0.177 | 0.989 | -1.962 | 0.013 | -0.196 | 0.021 |
| CAT | 0.026±1.365 | 1.188±1.900 | 0.762 | 0.020* | -0.304 | 3.283 | 0.011 | 0.364 |
| PL | -0.252±1.523 | 0.820±1.557 | 0.323 | 0.068 | -1.322 | 2.244 | -0.097 | 0.299 |

Supplementary Table 6: Table of between-subjects effects for ANCOVA analysis of contralateral amygdala subfields volume

| Source | Structural | df | F | P |
| --- | --- | --- | --- | --- |
| Corrected Model | whole_amy | 4 | 4.601 | 0.002 |
|  | La | 4 | 4.455 | 0.003 |
|  | Ba | 4 | 5.109 | 0.001 |
|  | AB | 4 | 3.432 | 0.012 |
|  | AAA | 4 | 3.095 | 0.020 |
|  | Ce | 4 | 4.719 | 0.002 |
|  | Me | 4 | 3.073 | 0.021 |
|  | Co | 4 | 2.408 | 0.056 |
|  | CAT | 4 | 4.468 | 0.003 |
|  | PL | 4 | 4.816 | 0.002 |
| Intercept | whole_amy | 1 | 1.775 | 0.186 |
|  | La | 1 | 1.955 | 0.166 |
|  | Ba | 1 | 2.030 | 0.158 |
|  | AB | 1 | 1.247 | 0.267 |
|  | AAA | 1 | 0.548 | 0.461 |
|  | Ce | 1 | 13.910 | <0.001 |
|  | Me | 1 | 6.949 | 0.010 |
|  | Co | 1 | 1.630 | 0.205 |
|  | CAT | 1 | 0.147 | 0.702 |
|  | PL | 1 | 2.314 | 0.132 |
| Sex | whole_amy | 1 | 2.168 | 0.145 |
|  | La | 1 | 2.501 | 0.118 |
|  | Ba | 1 | 1.374 | 0.244 |
|  | AB | 1 | 1.820 | 0.181 |
|  | AAA | 1 | 2.844 | 0.095 |
|  | Ce | 1 | 0.431 | 0.513 |
|  | Me | 1 | 0.747 | 0.390 |
|  | Co | 1 | 1.851 | 0.177 |
|  | CAT | 1 | 3.032 | 0.085 |
|  | PL | 1 | 1.075 | 0.303 |
| Age | whole_amy | 1 | 8.814 | 0.004 |
|  | La | 1 | 10.321 | 0.002 |
|  | Ba | 1 | 8.308 | 0.005 |
|  | AB | 1 | 6.483 | 0.013 |
|  | AAA | 1 | 4.217 | 0.043 |
|  | Ce | 1 | 12.389 | 0.001 |
|  | Me | 1 | 2.960 | 0.089 |
|  | Co | 1 | 5.041 | 0.027 |
|  | CAT | 1 | 1.812 | 0.182 |
|  | PL | 1 | 8.274 | 0.005 |
| Groups | whole_amy | 2 | 4.371 | 0.016 |
|  | La | 2 | 2.829 | 0.065 |
|  | Ba | 2 | 6.019 | 0.004 |
|  | AB | 2 | 3.282 | 0.042 |
|  | AAA | 2 | 3.650 | 0.030 |
|  | Ce | 2 | 4.272 | 0.017 |
|  | Me | 2 | 4.972 | 0.009 |
|  | Co | 2 | 2.328 | 0.104 |
|  | CAT | 2 | 7.029 | 0.002 |
|  | PL | 2 | 5.545 | 0.005 |
| Error | whole_amy | 83 | - | - |
|  | La | 83 | - | - |
|  | Ba | 83 | - | - |
|  | AB | 83 | - | - |
|  | AAA | 83 | - | - |
|  | Ce | 83 | - | - |
|  | Me | 83 | - | - |
|  | Co | 83 | - | - |
|  | CAT | 83 | - | - |
|  | PL | 83 | - | - |
| Total | whole_amy | 88 | - | - |
|  | La | 88 | - | - |
|  | Ba | 88 | - | - |
|  | AB | 88 | - | - |
|  | AAA | 88 | - | - |
|  | Ce | 88 | - | - |
|  | Me | 88 | - | - |
|  | Co | 88 | - | - |
|  | CAT | 88 | - | - |
|  | PL | 88 | - | - |
| Corrected Total | whole_amy | 87 | - | - |
|  | La | 87 | - | - |
|  | Ba | 87 | - | - |
|  | AB | 87 | - | - |
|  | AAA | 87 | - | - |
|  | Ce | 87 | - | - |
|  | Me | 87 | - | - |
|  | Co | 87 | - | - |
|  | CAT | 87 | - | - |
|  | PL | 87 | - | - |

Supplementary Table 7: Morphometric comparison of contralateral hippocampal subfields between MTLE patients and HC

|  | HYP _z-score_ | LVF _z-score_ | P-value _(HYP-HC)_ | P-value _(LVF-HC)_ | t _(HYP-HC)_ | t _(LVF-HC)_ | Cohen’s d _(HYP-HC)_ | Cohen’s d _(LVF-HC)_ |
| --- | --- | --- | --- | --- | --- | --- | --- | --- |
| Whole-hip | 0.102±1.621 | 0.489±1.157 | 0.883 | 0.224 | -0.428 | 1.512 | 0.038 | 0.221 |
| Parasubiculum | -0.571±1.081 | -0.382±1.490 | 0.754 | 0.269 | -1.921 | -1.335 | -0.264 | -0.149 |
| Presubiculum | -0.231±1.431 | 0.131±1.108 | 0.849 | 0.822 | -1.468 | 0.392 | -0.093 | 0.062 |
| Subiculum | 0.068±1.669 | 0.353±1.096 | 0.852 | 0.390 | -0.744 | 1.042 | 0.025 | 0.166 |
| CA1 | -0.054±1.423 | 0.747±1.362 | 0.852 | 0.117 | -0.893 | 2.245 | -0.022 | 0.298 |
| CA2/3 | 0.077±0.968 | 0.417±1.111 | 0.883 | 0.224 | -0.264 | 1.700 | 0.039 | 0.194 |
| CA4 | 0.191±1.454 | 0.488±1.282 | 0.883 | 0.224 | -0.148 | 1.616 | 0.076 | 0.208 |
| GC-DG | 0.134±1.480 | 0.517±1.311 | 0.883 | 0.224 | -0.300 | 1.640 | 0.053 | 0.216 |
| ML | -0.002±1.552 | 0.482±1.160 | 0.852 | 0.224 | -0.851 | 1.497 | -0.001 | 0.217 |
| HATA | 0.271±1.458 | 0.884±1.917 | 0.883 | 0.111 | 0.430 | 2.443 | 0.108 | 0.278 |
| Fimbria | -0.011±1.230 | 1.244±1.710 | 0.883 | 0.013* | -0.232 | 3.504 | -0.005 | 0.406 |
| Hip-fissure | -0.126±1.450 | 0.064±1.245 | 0.852 | 0.822 | -0.881 | 0.225 | -0.051 | 0.028 |
| Hip-tail | 0.461±1.278 | -0.073±1.014 | 0.849 | 0.822 | 1.305 | -0.261 | 0.197 | -0.036 |

Supplementary Table 8: Table of between-subjects effects for ANCOVA analysis of contralateral hippocampal subfields volume

| Source | Structural | df | F | P |
| --- | --- | --- | --- | --- |
| Corrected Model | Whole_hip | 4 | 4.924 | 0.001 |
|  | Parasubiculum | 4 | 1.061 | 0.381 |
|  | Presubiculum | 4 | 4.013 | 0.005 |
|  | Subiculum | 4 | 7.146 | <0.001 |
|  | CA1 | 4 | 5.431 | 0.001 |
|  | CA2/3 | 4 | 3.972 | 0.005 |
|  | CA4 | 4 | 5.747 | <0.001 |
|  | GC-DG | 4 | 4.908 | 0.001 |
|  | ML | 4 | 5.834 | <0.001 |
|  | HATA | 4 | 2.454 | 0.052 |
|  | Fimbria | 4 | 5.112 | 0.001 |
|  | Hip_fissure | 4 | 2.396 | 0.057 |
|  | Hip_tail | 4 | 1.832 | 0.130 |
| Intercept | Whole_hip | 1 | 0.425 | 0.516 |
|  | Parasubiculum | 1 | 1.218 | 0.273 |
|  | Presubiculum | 1 | 0.176 | 0.676 |
|  | Subiculum | 1 | 1.338 | 0.251 |
|  | CA1 | 1 | 2.252 | 0.137 |
|  | CA2/3 | 1 | 0.389 | 0.534 |
|  | CA4 | 1 | 0.015 | 0.903 |
|  | GC-DG | 1 | 0.121 | 0.728 |
|  | ML | 1 | 1.081 | 0.302 |
|  | HATA | 1 | 0.830 | 0.365 |
|  | Fimbria | 1 | 1.515 | 0.222 |
|  | Hip_fissure | 1 | 0.065 | 0.799 |
|  | Hip_tail | 1 | 0.140 | 0.709 |
| sex | Whole_hip | 1 | 7.803 | 0.006 |
|  | Parasubiculum | 1 | 0.000 | 0.985 |
|  | Presubiculum | 1 | 8.882 | 0.004 |
|  | Subiculum | 1 | 11.598 | 0.001 |
|  | CA1 | 1 | 2.616 | 0.110 |
|  | CA2/3 | 1 | 10.013 | 0.002 |
|  | CA4 | 1 | 11.929 | 0.001 |
|  | GC-DG | 1 | 8.758 | 0.004 |
|  | ML | 1 | 8.051 | 0.006 |
|  | HATA | 1 | 3.418 | 0.068 |
|  | Fimbria | 1 | 0.834 | 0.364 |
|  | Hip_fissure | 1 | 7.247 | 0.009 |
|  | Hip_tail | 1 | 2.808 | 0.098 |
| age | Whole_hip | 1 | 9.509 | 0.003 |
|  | Parasubiculum | 1 | 0.629 | 0.430 |
|  | Presubiculum | 1 | 6.249 | 0.014 |
|  | Subiculum | 1 | 16.214 | <0.001 |
|  | CA1 | 1 | 11.293 | 0.001 |
|  | CA2/3 | 1 | 3.184 | 0.078 |
|  | CA4 | 1 | 8.771 | 0.004 |
|  | GC-DG | 1 | 8.208 | 0.005 |
|  | ML | 1 | 12.435 | 0.001 |
|  | HATA | 1 | 0.539 | 0.465 |
|  | Fimbria | 1 | 2.359 | 0.128 |
|  | Hip_fissure | 1 | 2.271 | 0.136 |
|  | Hip_tail | 1 | 0.912 | 0.342 |
| groups | Whole_hip | 2 | 1.805 | 0.171 |
|  | Parasubiculum | 2 | 2.024 | 0.139 |
|  | Presubiculum | 2 | 1.674 | 0.194 |
|  | Subiculum | 2 | 1.370 | 0.260 |
|  | CA1 | 2 | 4.503 | 0.014 |
|  | CA2/3 | 2 | 2.002 | 0.142 |
|  | CA4 | 2 | 1.702 | 0.189 |
|  | GC-DG | 2 | 1.914 | 0.154 |
|  | ML | 2 | 2.412 | 0.096 |
|  | HATA | 2 | 3.182 | 0.047 |
|  | Fimbria | 2 | 7.817 | 0.001 |
|  | Hip_fissure | 2 | 0.595 | 0.554 |
|  | Hip_tail | 2 | 1.233 | 0.297 |
| Error | Whole_hip | 83 | - | - |
|  | Parasubiculum | 83 | - | - |
|  | Presubiculum | 83 | - | - |
|  | Subiculum | 83 | - | - |
|  | CA1 | 83 | - | - |
|  | CA2/3 | 83 | - | - |
|  | CA4 | 83 | - | - |
|  | GC-DG | 83 | - | - |
|  | ML | 83 | - | - |
|  | HATA | 83 | - | - |
|  | Fimbria | 83 | - | - |
|  | Hip_fissure | 83 | - | - |
|  | Hip_tail | 83 | - | - |
| Total | Whole_hip | 88 | - | - |
|  | Parasubiculum | 88 | - | - |
|  | Presubiculum | 88 | - | - |
|  | Subiculum | 88 | - | - |
|  | CA1 | 88 | - | - |
|  | CA2/3 | 88 | - | - |
|  | CA4 | 88 | - | - |
|  | GC-DG | 88 | - | - |
|  | ML | 88 | - | - |
|  | HATA | 88 | - | - |
|  | Fimbria | 88 | - | - |
|  | Hip_fissure | 88 | - | - |
|  | Hip_tail | 88 | - | - |
| Corrected Total | Whole_hip | 87 | - | - |
|  | Parasubiculum | 87 | - | - |
|  | Presubiculum | 87 | - | - |
|  | Subiculum | 87 | - | - |
|  | CA1 | 87 | - | - |
|  | CA2/3 | 87 | - | - |
|  | CA4 | 87 | - | - |
|  | GC-DG | 87 | - | - |
|  | ML | 87 | - | - |
|  | HATA | 87 | - | - |
|  | Fimbria | 87 | - | - |
|  | Hip_fissure | 87 | - | - |
|  | Hip_tail | 87 | - | - |

Supplementary Table 9: Metabolic comparison of ipsilateral amygdala and hippocampal subfields between MTLE patients and HC

|  | HYP _z-score_ | LVF _z-score_ | P-value _(HYP-HC)_ | P-value _(LVF-HC)_ | t _(HYP-HC)_ | t _(LVF-HC)_ | Cohen’s d _(HYP-HC)_ | Cohen’s d _(LVF-HC)_ |
| --- | --- | --- | --- | --- | --- | --- | --- | --- |
| Whole-amy | -1.585±6.009 | -1.557±2.422 | 0.296 | 0.505 | -1.194 | -1.039 | -0.181 | -0.387 |
| Whole-hip | -2.917±2.874 | -0.656±3.510 | 0.013* | 0.795 | -2.939 | -0.325 | -0.561 | -0.126 |
| Head | -3.692±4.577 | -1.575±4.126 | 0.013* | 0.505 | -2.937 | -1.182 | -0.487 | -0.254 |
| Body | -2.365±2.727 | -0.167±2.937 | 0.018* | 0.795 | -2.619 | 0.261 | -0.499 | -0.038 |
| Tail | -1.237±3.222 | 0.821±3.226 | 0.452 | 0.340 | -0.757 | 1.857 | -0.251 | 0.169 |

Supplementary Table 10: Table of between-subjects effects for ANCOVA analysis of ipsilateral amygdala and hippocampal subfields SUVR

| Source | Structural | df | F | P |
| --- | --- | --- | --- | --- |
| Corrected Model | whole_amy | 4 | 0.757 | 0.557 |
|  | whole_hip | 4 | 4.654 | 0.002 |
|  | hip_head | 4 | 2.935 | 0.027 |
|  | hip_body | 4 | 4.871 | 0.002 |
|  | hip_tail | 4 | 4.506 | 0.003 |
| Intercept | whole_amy | 1 | 0.288 | 0.594 |
|  | whole_hip | 1 | 7.572 | 0.008 |
|  | hip_head | 1 | 3.269 | 0.075 |
|  | hip_body | 1 | 8.151 | 0.006 |
|  | hip_tail | 1 | 7.747 | 0.007 |
| sex | whole_amy | 1 | 0.339 | 0.563 |
|  | whole_hip | 1 | 0.852 | 0.360 |
|  | hip_head | 1 | 0.404 | 0.527 |
|  | hip_body | 1 | 1.165 | 0.284 |
|  | hip_tail | 1 | 0.272 | 0.604 |
| age | whole_amy | 1 | 0.368 | 0.546 |
|  | whole_hip | 1 | 3.617 | 0.062 |
|  | hip_head | 1 | 0.639 | 0.427 |
|  | hip_body | 1 | 4.419 | 0.040 |
|  | hip_tail | 1 | 10.185 | 0.002 |
| groups | whole_amy | 2 | 0.810 | 0.449 |
|  | whole_hip | 2 | 5.624 | 0.006 |
|  | hip_head | 2 | 4.479 | 0.015 |
|  | hip_body | 2 | 5.647 | 0.006 |
|  | hip_tail | 2 | 4.112 | 0.021 |
| Error | whole_amy | 63 | - | - |
|  | whole_hip | 63 | - | - |
|  | hip_head | 63 | - | - |
|  | hip_body | 63 | - | - |
|  | hip_tail | 63 | - | - |
| Total | whole_amy | 68 | - | - |
|  | whole_hip | 68 | - | - |
|  | hip_head | 68 | - | - |
|  | hip_body | 68 | - | - |
|  | hip_tail | 68 | - | - |
| Corrected Total | whole_amy | 67 | - | - |
|  | whole_hip | 67 | - | - |
|  | hip_head | 67 | - | - |
|  | hip_body | 67 | - | - |
|  | hip_tail | 67 | - | - |

Supplementary Table 11: Metabolic comparison of contralateral amygdala and hippocampus subfields between MTLE patients and HC

|  | HYP _z-score_ | LVF _z-score_ | P-value _(HYP-HC)_ | P-value _(LVF-HC)_ | t _(HYP-HC)_ | t _(LVF-HC)_ | Cohen’s d _(HYP-HC)_ | Cohen’s d _(LVF-HC)_ |
| --- | --- | --- | --- | --- | --- | --- | --- | --- |
| Whole-amy | 1.043±4.989 | 0.161±3.310 | 0.391 | 0.819 | 0.863 | 0.229 | 0.143 | 0.033 |
| Whole-hip | 1.438±3.514 | 2.111±4.077 | 0.220 | 0.042* | 1.610 | 2.302 | 0.268 | 0.335 |
| Head | 0.942±4.700 | 0.985±3.960 | 0.391 | 0.408 | 0.887 | 0.990 | 0.137 | 0.168 |
| Body | 1.137±3.350 | 2.111±3.500 | 0.220 | 0.033* | 1.527 | 2.567 | 0.224 | 0.379 |
| Tail | 2.768±3.840 | 3.554±4.010 | 0.030* | 0.005* | 2.847 | 3.661 | 0.442 | 0.520 |

Supplementary Table 12: Table of between-subjects effects for ANCOVA analysis of contralateral amygdala and hippocampal subfields SUVR

| Source | Structural | df | F | P |
| --- | --- | --- | --- | --- |
| Corrected Model | whole_amy | 4 | 0.483 | 0.748 |
|  | whole_hip | 4 | 1.395 | 0.246 |
|  | hip_head | 4 | 0.301 | 0.876 |
|  | hip_body | 4 | 1.848 | 0.131 |
|  | hip_tail | 4 | 3.582 | 0.011 |
| Intercept | whole_amy | 1 | 0.666 | 0.417 |
|  | whole_hip | 1 | 0.014 | 0.907 |
|  | hip_head | 1 | 0.023 | 0.880 |
|  | hip_body | 1 | 0.316 | 0.576 |
|  | hip_tail | 1 | 0.135 | 0.714 |
| sex | whole_amy | 1 | 0.842 | 0.362 |
|  | whole_hip | 1 | 0.004 | 0.952 |
|  | hip_head | 1 | 0.074 | 0.786 |
|  | hip_body | 1 | 0.226 | 0.636 |
|  | hip_tail | 1 | 0.015 | 0.902 |
| age | whole_amy | 1 | 0.002 | 0.964 |
|  | whole_hip | 1 | 0.889 | 0.349 |
|  | hip_head | 1 | 0.187 | 0.667 |
|  | hip_body | 1 | 1.442 | 0.234 |
|  | hip_tail | 1 | 1.127 | 0.293 |
| groups | whole_amy | 2 | 0.421 | 0.658 |
|  | whole_hip | 2 | 2.721 | 0.074 |
|  | hip_head | 2 | 0.569 | 0.569 |
|  | hip_body | 2 | 3.300 | 0.043 |
|  | hip_tail | 2 | 7.150 | 0.002 |
| Error | whole_amy | 63 | - | - |
|  | whole_hip | 63 | - | - |
|  | hip_head | 63 | - | - |
|  | hip_body | 63 | - | - |
|  | hip_tail | 63 | - | - |
| Total | whole_amy | 68 | - | - |
|  | whole_hip | 68 | - | - |
|  | hip_head | 68 | - | - |
|  | hip_body | 68 | - | - |
|  | hip_tail | 68 | - | - |
| Corrected Total | whole_amy | 67 | - | - |
|  | whole_hip | 67 | - | - |
|  | hip_head | 67 | - | - |
|  | hip_body | 67 | - | - |
|  | hip_tail | 67 | - | - |
